# Pyruvate Substitutions on Glycoconjugates

**DOI:** 10.3390/ijms20194929

**Published:** 2019-10-05

**Authors:** Fiona F. Hager, Leander Sützl, Cordula Stefanović, Markus Blaukopf, Christina Schäffer

**Affiliations:** 1 Department of NanoBiotechnology, NanoGlycobiology unit, Universität für Bodenkultur Wien, Muthgasse 11, A-1190 Vienna, Austria; fiona.hager@boku.ac.at (F.F.H.); cordula.brinskele@boku.ac.at (C.S.); christina.schaeffer@boku.ac.at (C.Sch.); 2 Department of Food Science and Technology, Food Biotechnology Laboratory, Muthgasse 11, Universität für Bodenkultur Wien, A-1190 Vienna, Austria; leander.suetzl@boku.ac.at; 3 Department of Chemistry, Division of Organic Chemistry, Universität für Bodenkultur Wien, Muthgasse 18, A-1190 Vienna, Austria; markus.blaukopf@boku.ac.at

**Keywords:** pyruvylation, pyruvyltransferase, exopolysaccharides, capsular polysaccharides, cell wall glycopolymers, *N*-glycans, lipopolysaccharides, biosynthesis, sequence space, pyruvate analytics

## Abstract

Glycoconjugates are the most diverse biomolecules of life. Mostly located at the cell surface, they translate into cell-specific “barcodes” and offer a vast repertoire of functions, including support of cellular physiology, lifestyle, and pathogenicity. Functions can be fine-tuned by non-carbohydrate modifications on the constituting monosaccharides. Among these modifications is pyruvylation, which is present either in enol or ketal form. The most commonly best-understood example of pyruvylation is enol-pyruvylation of *N*-acetylglucosamine, which occurs at an early stage in the biosynthesis of the bacterial cell wall component peptidoglycan. Ketal-pyruvylation, in contrast, is present in diverse classes of glycoconjugates, from bacteria to algae to yeast—but not in humans. Mild purification strategies preventing the loss of the acid-labile ketal-pyruvyl group have led to a collection of elucidated pyruvylated glycan structures. However, knowledge of involved pyruvyltransferases creating a ring structure on various monosaccharides is scarce, mainly due to the lack of knowledge of fingerprint motifs of these enzymes and the unavailability of genome sequences of the organisms undergoing pyruvylation. This review compiles the current information on the widespread but under-investigated ketal-pyruvylation of monosaccharides, starting with different classes of pyruvylated glycoconjugates and associated functions, leading to pyruvyltransferases, their specificity and sequence space, and insight into pyruvate analytics.

## 1. Introduction

**Figure 1:**
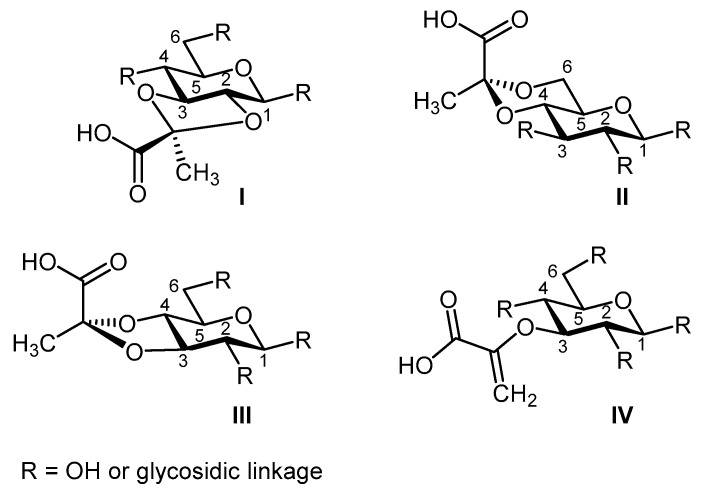
Overview on the most common modes of monosaccharide pyruvylation. Shown is pyruvate ketal at bridging positions 2,3 (**I**), 4,6 (**II**), and 3,4 (**III**), and enol pyruvate (**IV**). Arabic numbers indicate ring positions.

Pyruvylation is a widespread non-carbohydrate modification of monosaccharides found in various classes of glycoconjugates. In most cases, the modification is present as a pyruvate (Pyr) ketal (cyclic acetal/ketal) bridging two hydroxyl groups of a monosaccharide residue and forming a ring structure [1], where pyruvate is most frequently placed across the 2,3-, 4,6-, or 3,4-positions (Figure 1I–III). The best-known example of pyruvylation, however, occurs as enol pyruvate (Figure 1IV), which is elaborated during the biosynthesis of the bacterial cell wall component peptidoglycan [2]. In both modes of pyruvylation, a dedicated pyruvyltransferase catalyses the transfer of the pyruvate moiety to the monosaccharide target.

Pyruvate-ketal-modified (henceforth abbreviated as “pyruvylated”) glycoconjugates are found in various phylogenetic orders of life, including bacteria, yeast, and algae, but not in humans. Pyruvylated glycoconjugates are typically present in the cell envelope to which they impart a net negative charge; this is necessary for vital biological functions, such as regulation of the cell influx/efflux processes and cell–cell interactions including cell aggregation and pathogenic adhesion. Notably, besides pyruvylation, nature offers various alternate strategies to create anionic cell surfaces, including a wide range of acidic saccharides (e.g., muramic acid, hexuronic acids, sialic acids) and saccharide modifications (e.g., succinate, lactate, phosphate) [3]. These compounds further lead to an increased capability of cells to electrostatically bind cations at the surface, which, in turn, may foster the packing density of the saccharide portion of glycoconjugates [4].

The repertoire of monosaccharide targets of pyruvylation is quite diverse. The most abundant pyruvylated monosaccharide with 59 hits in the Carbohydrate Structure Database (CSDB, http://csdb.glycoscience.ru/) [5,6] is galactose (Gal). Examples of pyruvylated galactose include, among others, the capsular polysaccharide of *Bacteroides fragilis* [7] and *Streptococcus pneumoniae* [8], *N*-glycans of the fission yeast *Schizosaccharomyces pombe* [9], as well as carragenans [10], and galactans from algae [11,12,13]. Recently, pyruvylated *N*-acetylmannosamine (ManNAc) has emerged as an important epitope on bacterial “non-classical” secondary cell wall glycopolymers, serving as a cell wall ligand for cell surface (S-) layer proteins, such as those of the pathogen *Bacillus anthracis* and the honeybee saprophyte *Paenibacillus alvei* [14,15]. There are also examples of pyruvylated monosaccharides on capsular polysaccharides serving as an immunostimulatory effector [7,16], or contributor to virulence as in the case of the secondary cell wall polymer of *Bacillus cereus* [17] or the exopolysaccharide xanthan of *Xanthomonas* spp., where pyruvylation is essential for successful colonization and pathogenesis in planta [18]. Pyruvyl groups on terminal glucose (Glc) and *N*-acetylgalactosamine (GalNAc) residues in the lipooligosaccharide of *Pseudomonas stutzeri* OX1, in contrast, are assumed to have biosynthetic implications [4]. All these examples are, among others, discussed in detail below.

Several studies dealing with pyruvylated glycans and their structural elucidation are available in the literature. However, knowledge of the enzymatic machinery governing pyruvylation is scarce. This is mainly due to missing sequencing data of the organisms, which produce pyruvylated glycoconjugates of known structure. Thus, despite their predictably widespread occurrence, pyruvyltransferases are an under-investigated class of enzymes.

This review summarizes the current state of knowledge about pyruvylated glycoconjugates in nature—focusing on bacterial sources—with an emphasis on the pyruvyltransferases involved in their biosynthesis.

## 2. Enol-Pyruvylation in Peptidoglycan Biosynthesis

The UDP-*N-*acetylglucosamine-3-*O*-enol-pyruvyltransferase MurA (EC 2.5.1.7) targets UDP-*N*-acetylglucosamine (UDP-GlcNAc) as an acceptor substrate for enol-pyruvyl transfer from a phosphoenolpyruvate (PEP) substrate, thereby releasing free phosphate and yielding the UDP-activated form of the essential bacterial cell wall compound *N*-acetylmuramic acid (enolpyruvyl-UDP-*N*-acetylglucosamine; MurNAc). This first committed step of peptidoglycan biosynthesis is inhibited by the epoxide antibiotic fosfomycin [19]. As a PEP analogue, fosfomycin binds covalently to the key cysteine residue at position 115/116 (position depending on the source of enzyme) in the active site of MurA, preventing the formation of UDP-MurNAc [19,20,21]. The co-crystal structure of a Cys (cysteine)-to-Ser (serine) mutant of *Enterobacter cloacae*, MurA, together with its substrates, revealed that the Cys residue is essential for product release and not directly involved in the chemical reaction of enol-pyruvyl transfer. The comparison of the product state with the intermediate state and an unliganded state of MurA indicated that the dissociation of the products is an ordered event, with inorganic phosphate leaving first, followed by conformational changes that lead to the opening of the two-domain structure of MurA and the final release of UDP-MurNAc [20]. A recent study on MurA of the opportunistic pathogen *Acinetobacter baumannii* revealed that the enzyme exists as a monomer in solution and has a pH optimum of 7.5 at 37 °C. The Km for UDP-GlcNAc is 1.062 ± 0.09 mM and 1.806 ± 0.23 mM for PEP [21]. The relative enzymatic activity is inhibited approximately threefold in the presence of 50 mM fosfomycin. Superimposition of a model for the *A. baumannii* enzyme with MurA of *Escherichia coli (E. coli)* confirmed the structural similarity in the fosfomycin binding site. Because of the worldwide spread of antimicrobial resistance and the paucity of novel drugs in the development pipeline, there has been a renewed interest in fosfomycin as an alternative option for the treatment of infections caused by multidrug-resistant Gram-negative bacteria [22]. However, it has to be considered that natural MurA mutants exist that render the respective organisms fosfomycin resistant. This includes *Mycobacteria* and *Chlamydia* species, where Cys-to-Asp mutants occur [20].

Interestingly, NikO, another enol-pyruvyltransferase that is structurally closely related to the common MurA enzymes and, consequently, inhibited by fosfomycin, plays an essential role in the biosynthesis of nikkomycins. Nikkomycins are peptide-nucleoside antibiotics, which strongly inhibit chitin synthesis and, therefore, are effective against fungi and insects. NikO was shown to transfer the enol-pyruvyl moiety from PEP to the 3′-hydroxyl group of UMP and to be inactivated by fosfomycin because of alkylation of Cys130. However, the degree of inactivation is not as pronounced as in the case of common MurA enzymes [23].

## 3. Ketal-Pyruvylated Glycoconjugates

In this section, an overview of the different classes of pyruvylated glycoconjugates is given, including glycan composition and structure, as well as functional and biosynthetic aspects, when known.

There is a recent interest in understanding the biosynthetic pathways of pyruvylated glycoconjugates from bacterial sources, as these pathways might unravel novel targets for therapeutic intervention. However, the current biosynthesis models for the different classes of glycoconjugates are in most cases only fragmentarily available and, frequently, they consider in silico predictions of involved components without experimental evidence.

### 3.1. Exopolysaccharides

Many organisms produce extracellular polysaccharides (exopolysaccharide, EPS) that are actively secreted during growth, including bacteria, yeasts, and microalgae [24]. EPSs are a diverse class of carbohydrate polymers that are composed of either linear or branched repeating units that are connected with varying stereochemistry. Monosaccharide constituents include pentoses (ribose and arabinose—especially in *Mycobacterium* spp.), hexoses (mannose (Man), glucose, fructose, galactose), deoxysugars (rhamnose (Rha), fucose (Fuc), uronic acids (glucuronic and galacturonic acids), and amino sugars (glucosamine, galactosamine, in several cases modified by *N*-acetylation) [24]. Depending on the monosaccharide composition, homo- or hetero-polymers are differentiated.

EPSs have a “jelly-like” appearance and are part of the glycocalyx—with which the “cellular sugar coat” is referred to [24]; as a common feature, they create a protective matrix around cells. The shielding effect against macromolecules that is conferred by EPS makes some bacteria 1000 times more resistant to antibiotics than their EPS-free counterparts [25].

Given the high application potential of microbial EPSs in medical fields, biomaterials, food applications, and in the replacement of petro-based chemicals [26], these glycoconjugates are currently of high interest.

#### 3.1.1. Xanthan

Xanthan is the main EPS produced by *Xanthomonas campestris* and other phytopathogenic *Xanthomonas* spp. that cause various economically important diseases in mono- and di-cotyledonous crops. Xanthan enhances the attachment to plant surfaces through its effect on biofilm formation, promotes pathogenesis by Ca^2+^ chelation and, thereby, suppression of the plant defence responses in which Ca^2+^ acts as a signal [27]. In practical applications, xanthan is frequently used as a viscosifying agent [28,29].

The pentasaccharide-repeating unit of xanthan consists of two β-(1→4) linked d-Glc residues as backbone and a trisaccharide side chain, α-(1→3)-linked to every other glucose. The side chain is composed of α-d-Man, β-d-glucuronic acid (GlcA), and β-d-Man, which are β-(1→2)- and β-(1→4)-linked to another, respectively [30]. In its natural state, the α-d-Man residue is acetylated and the β-d-Man is either acetylated or pyruvylated. It was found that the 4,6-ketal-pyruvate (4,6Pyr) specifically and, to a lesser extent, the acetyl groups that decorate the mannose residues are involved in Ca^2+^ chelation [27] and affect bacterial adhesion and biofilm architecture and, hence, contribute to the bacterium’s virulence [18]. Furthermore, the rheological properties of xanthan are influenced by its pyruvylation and acetylation pattern [28,31].

Xanthan biosynthesis is encoded in a so-called *gum*-cluster. Of the 13 encoded genes, *gumDMHK* are involved in the synthesis of the pentasaccharide repeat, and *gumBCEJ* in polymerization and xanthan export across the outer membrane in a flippase/polymerase (Wzx/Wzy)-dependent pathway. Regarding the modifications of xanthan, the predicted pyruvyltransferase GumL is hypothesised to catalyse pyruvylation of β-d-Man residues, while GumF and GumG are involved in β-d-Man acetylation [28,32]. It remains to be determined at which stage of xanthan biosynthesis the modifications are elaborated; it might be either at the cytoplasmic membrane or in the periplasmic space [32]. To this end, it was shown that GumK, a glucuronic acid transferase, is active on the lipid-linked trisaccharide precursor α-Man-(1→3)-β-Glc-(1→4)-β-Glc-P-P-polyisoprenyl, and shows reduced activity on the acetylated precursor substrate 6-*O*-acetyl-α-Man-(1→3)-β-Glc-(1→4)-β-Glc-PP-polyisoprenyl [33]. This suggests that mannose acetylation occurs after the completion of the trisaccharide side chain [32,33]; this might also hold true for pyruvylation. The xanthan biosynthetic enzymes seem to be highly conserved among different organisms, except for the mannose-transferase GumI and the pyruvyltransferase GumL, for which no homologues are found in other organisms [34].

Interestingly, *Bacillus* sp. strain GL1, which utilizes xanthan for its growth, produces an extra- cellular xanthan lyase, which catalyses the cleavage of the glycosidic bond between 4,6Pyr-β-d-Man and β-d-GlcA residues in xanthan side chains and, thus, contributes to depolymerisation of xanthan [35,36].

#### 3.1.2. Succinoglycan

Succinoglycan is a pyruvylated EPS that is produced by *Agrobacterium* [37,38], *Alcaligenes*, *Pseudomonas* [39], and *Rhizobium* strains [40], and is of great importance in plant symbiosis.

**Figure 2:**
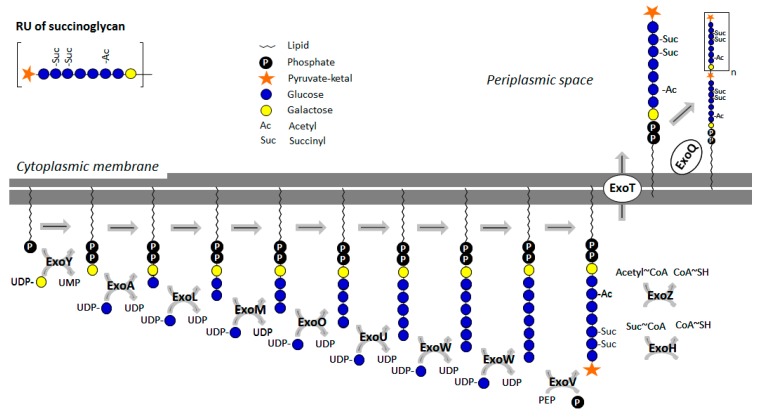
Scheme of succinoglycan biosynthesis in *Shinorhizobium meliloti* [43]. The pyruvylation step occurs in the cytoplasm at the stage of the undp-PP-linked RU prior to export and polymerization in the periplasmic space. Pyruvylation (ExoV) is indicated by a star. The order of pyruvylation, acetylation (ExoZ), and succinylation (Exo) is unknown. RU: repeating unit. Monosaccharide symbols are shown according to the Symbol Nomenclature for Glycans (SNFG) [45].

It is a heteropolymer that is multiply decorated with pyruvate, succinate, and acetate substituents. While the extent of acetylation and succinylation depends on the strain and the cultivation conditions, pyruvate is always found in a stoichiometric manner at the terminal β-Glc residue [32]. The repeat unit structure of succinoglycan is composed of β-Glc and β-Gal in a molar ratio of 7:1. Nineteen genes are involved in the polymer’s biosynthesis, which are referred to as *exo* genes and encoded in a 16 kb gene cluster. The biosynthesis starts with the production of the nucleotide-activated sugars UDP-Glc and UDP-Gal, where ExoC (phosphoglucomutase), ExoB (UDP-glucose-4-epimerase), and ExoN (UDP-pyrophosphorylase) are involved [32,41,42] (Figure 2). The initial step in the biosynthesis is executed by ExoY, a priming galactosyltransferase transferring a single, reducing-end Gal residue onto an undecaprenylphosphate (undp-P) carrier. *ExoA*, *exoL*, *exoM*, *exoO*, *exoU*, and *exoW* encode subsequent glycosyltransferases, which complete the octa-saccharide repeat in a step-wise manner, with each enzyme transferring a single monosaccharide, each, except for ExoW which transfers the subterminal and terminal glucoses. Prior to the export of the octasaccharide via a Wzx-dependent pathway, pyruvylation (at the terminal, non- reducing-end glucose), acetylation, and succinylation reactions catalysed by ExoV, ExoZ, and ExoH, handed over to ExoQ, which is responsible for polymerization of the fully modified repeats [43]. Studies on ExoV, the pyruvyltransferase of *Shinorhizobium* (previously *Rhizobium*) *meliloti*, suggest that pyruvylation is important for polymerization of repeating units and efficient succinoglycan export [44].

For *Rhizobium leguminosarum*, it was shown that missing pyruvylation on the terminal glucose residue of the succinoglycan impairs the formation of the nitrogen-fixing symbiosis with *Pisum sativum*, supportive of a signalling role of pyruvylation in this process [46]. PssK was identified as the pyruvyltransferase involved in succinoglycan modification of *R. leguminosarum* [46].

#### 3.1.3. Salecan

The salt-tolerant soil bacterium *Agrobacterium* sp. ZX09 is the producer of salecan, a soluble, succinylated, and pyruvylated EPS with a β-(1→3) glucan structure that is of interest because of its multiple bioactivities and unusual rheological properties. Its basic repeating unit structure was initially elucidated as →3)-β-d-Glc*p*-(1→3)-[β-d-Glc*p*-(1→3)-β-d-Glc*p*-(1→3)]-α-d-Glc*p*-(1→3)-α-d-Glc*p*-(1→ [47]. On the basis of amino acid homology with the respective *exo* genes, it can be concluded that succinyl- and pyruvyl-groups are conferred to salecan upon catalysis of SleA (succinyl-transferase) and SleV (pyruvyltransferase), respectively, both of which are located in a 19.6-kb gene cluster [48]. The exact positions of the salecan modifications remain to be determined.

#### 3.1.4. Colonic Acid

Colonic acid (CA) or M-antigen is another class of pyruvylated EPS mostly found in *Enterobacteriaceae*, including the majority of *Escherichia coli* strains. CA forms a loosely associated saccharide mesh that coats the bacteria, often within biofilms. CA is composed of hexasaccharide repeat units consisting of glucose, two fucoses, two galactoses, and glucuronic acid [49]. Additionally, acetylation is found on fucose or/and galactose, while pyruvylation is found on the terminal galactose only, with both modifications occurring non-stoichiometrically [32,50]. The overall structure of CA is 4,6Pyr-α-Gal*p*-(1→4)-β-Glc*p*A-(1→3)-*O*Ac-α-Gal*p*-(1→3)-α-Fuc*p*-(1→4)-*O*Ac-*O*Ac-α-Fuc*p*-(1→3)-β-Glc-(1→7)-α-Hep*p*-(1→6)-α-Glc*p*-(1→2)-α-Glc*p*-(1→3)-[α-Gal*p*-(1→6]-α-Glc*p*-(1→3)-[α-Hep*pP*-(1→7)]-α-Hep*pP*-(1→3)-[PEtN]α-Hep*p*-(1→5)-αKdo*p*-(1→, where Kdo is 3-deoxy-d-*manno*-oct-2-ulosonic acid.

The genetic determinants for CA biosynthesis reside in a 19-gene *wca* (*cps*) cluster and are tightly regulated by a complex signal transduction cascade [51]. The gene cluster encodes six glycosyl-transferases, named WcaJ, WcaI, WcaE, WcaC, WcaL, and WcaA. Furthermore, a putative pyruvyl-transferase (WcaK) is encoded next to two predicted acetyltransferases (WcaF and WcaB), although there are up to three acetylation positions described in CA [50]. Interestingly, WcaF seems to contribute to biofilm formation of the bacterium, since knocking out of this enzyme led to biofilm disruption under *in vitro* conditions [52].

#### 3.1.5. Unclassified Pyruvylated EPS

Up to now, there are several unclassified types of pyruvylated EPSs. The repeating unit structure of the acidic EPS produced by a mucoid strain of *Burkholderia cepacia* isolated from a cystic fibrosis patient was established as →3)-β-d-Gal*p*-(1→3)-4,6Pyr-α-Gal*p*-(1→ [53].

The freshwater biofilm isolate *Pseudomonas* strain 1.15 produces considerable amounts of an acidic EPS that is composed of repeating units with the structure →4)-[4,6Pyr-α-d-Gal*p*-(1→4)-β-d-GlcA*p*-(1→3)-α-d-Gal*p*-(*O*→3]-α-l-Fuc*p*-(1→4)-α-l-Fuc*p*-(1→3)-β-d-Glc*p*-(1→. Furthermore, of the four different *O*-acetyl groups present in non-stoichiometric amounts, two were established to be on O-2 of the 3-linked galactose and on O-2 of the 4-linked fucose [54].

*Enterobacter amnigenus*, a bacterium isolated from sugar beets, produces an EPS that is rich in l-Fuc and has a terminal, pyruvylated α-d-Man residue [55].

The cystic fibrosis lung pathogen *Inquilinus limosus* produces two EPSs with unique structures—an α-(1→2)-linked mannan and a β-(1→3)-linked glucan—both fully substituted with 4,6-linked pyruvate ketals [56,57]. Cystic fibrosis is an autosomal recessive disorder and its mortality is due to chronic microbial colonisation of the major airways that leads to exacerbation of pulmonary infection [58]. While *Pseudomonas aeruginosa* is one of the most threatening microbes colonizing cystic fibrosis lungs, the EPS of *I*. *limosus* is suspected to play a role in the pathogenesis of the disease.

The bacterium *Azorhizobium caulinodans* produces a linear homopolysaccharide-type EPS composed of α-(1→3)-linked 4,6Pyr-d-Gal residues. The bacterium undergoes a symbiotic interaction with *Sesbania rostrata* as a legume host plant, which results in the development of root nodules, accompanied by a massive production of H_2_O_2_. In situ H_2_O_2_ localization demonstrated that increased EPS production during early stages of invasion prevents the incorporation of H_2_O_2_ inside the bacteria, suggesting a role for EPS in protecting the microsymbiont against H_2_O_2_ [59].

A special K-antigen-like EPS is found in the marine bacterium *Cobetia marina* DSMZ 4741, with its repeating unit composed of ribose and pyruvylated Kdo [60].

Within the EPS structure of the lactic acid bacterium *Pediococcus pentosaceus* LP28, a pyruvate modification was described to occur on one of the four constituting monosaccharides (Glc, Gal, Man, and GlcNAc) [61]. The EPS biosynthetic gene cluster consists of 12 ORFs containing a priming enzyme, five glycosyltransferases, and a putative polysaccharide: pyruvyltransferase [61].

EPSs produced by an *Erwinia* spp. in association with the bacterium *Coniothyrium zuluense* are linked to a fungal canker disease of *Eucalyptus* [62]. One of these EPSs is that of *Erwinia stewartii*; another is that of *Erwinia futululu*, whose structures are identical except for the replacement of one terminal Glc residue by 4,6Pyr-Gal*p* in the latter, yielding →3)-β-d-Gal*p*-(1→3)[4,6Pyr-α-d-Gal*p*-(1→4)-β-d-Glc*p*A-(1→4)][β-d-Glc*p*-(1→6)]-α-d-Gal*p*-(1→6)-β-d-Glcp-(1→ [63].

*Agrobacterium radiobacter* (ATCC 53271) produces an anionic EPS that gives aqueous dispersions, exhibiting high viscosity at low concentrations. The *A. radiobacter* EPS is composed of a complex heptadekasaccharide repeating unit, which exposes a subterminal 4,6Pyr-α-d-Glc residue on each of the two identical tetraglycosyl branches [64].

*Methylobacterium* sp. is a slime-forming bacterium isolated from a Finnish paper machine, which is a high EPS producer. Its EPS repeating unit has the structure →3)-4,6Pyr-α-d-Gal*p*-(1→3)-4,6Pyr-α-d-Gal*p*-(1→3)-α-d-Gal*p*-(1→ [65].

The marine bacterium *Alteromonas macleodii* subsp. *fijiensis* isolated from deep-sea hydrothermal vents displays a pyruvylated mannose in its EPS hexasaccharide repeating unit structure →4)-β-d-Glc*p*-(1→4)[4,6Pyr-β-d-Manl*p*-(1→4)-β-d-Glc*p*A-(1→3)-α-d-Glc*p*A-(1→3)]α-d-Gal*p*A-(1→4)-α-d-Gal*p*-(1→ [66]. Aside from its use in the food industry, this marine polymer has been suggested to be used for the treatment of cardiovascular diseases and bone healing [67].

### 3.2. Capsular Polysaccharides

Capsular polysaccharides (CPSs) are also part of the glycocalyx but, in contrast to EPSs, are covalently connected to the bacterial cell surface via membrane phospholipids [24]. Because of their prominent cellular localization, CPSs are the first interaction zone of bacteria with the host immune system, and thus are important virulence factors of many bacteria. Very often, encapsulated bacteria are pathogenic, whereas capsule-deficient isolates are not [68]. Hence, CPSs are frequently used for the production of polysaccharide conjugate vaccines [69].

Bacterial capsules are formed primarily from long-chain polysaccharides with repeat-unit structures. A given bacterial species can produce a range of CPSs with different structures, and these aid in distinguishing isolates by serotyping [68]. The widespread occurrence and the high structural differences of CPSs are reflected by 84 capsular serotypes (K-antigens) found alone in *E. coli* strains. Essentially, there are four groups of capsules [70]. Group I- and IV-CPS, which are often found in organisms leading to gastrointestinal diseases, use the Wzx/Wzy-dependent export pathway and their biosynthesis proceeds on a polyprenol linker. Capsules from groups II and III use the ABC-transporter export pathway and are frequently present in mucosal pathogens such as *Neisseria meningitidis*. Interestingly, a CPS attached via a novel β-linked poly-3-deoxy-d-*manno*-oct-2-ulosonic acid linker to the phospholipid *lyso*-phosphatidylglycerol is present, which in earlier studies was described as a diacylglycerol because of hydrolysis experiments [68].

#### 3.2.1. *Streptococcus pneumoniae* CPS

*Streptococcus pneumoniae* (pneumococcus) is a leading cause of bacterial-induced pneumonia, meningitis, and bacteraemia globally [71]. Prevnar 13, the most broadly protective pneumococcal conjugate vaccine, is composed of 13 protein-polysaccharide conjugates consisting of pneumococcal CPS serotypes 1, 3, 4, 5, 6A, 6B, 7F, 9V, 14, 18C, 19A, 19F, and 23F—each individually linked to the genetically inactivated diphtheria toxoid CRM_197_. Currently, approaches on the basis of biocon-jugation and glycosylation engineering are being pursued as manufacturing alternatives to enable the production of vaccines with higher protection rates, especially in children [69,72,73].

*S. pneumonia* CPS serotype 4 (ST4) is a prevalent serotype in vaccine formulations, containing 2,3-pyruvate ketal on the Gal residue of its repeating units—with the structure →3)-β-d-Man*p*NAc-β-(1→3)-α-l-Fuc*p*NAc-(1→3)-Gal*p*NAc-α-(1→4)-2,3Pyr-α-d-Gal-(1→—which has been shown to be the key component of its specific immunogenic motif [74].

Thus, the pyruvate modification is essential for designing minimal synthetic carbohydrate vaccines for ST4, as vaccine formulations without pyruvylation would not recognize the natural CPS [8]. It is, therefore, highly recommended to include the pyruvate ketal epitope in glycoconjugate vaccines [16].

#### 3.2.2. *Acinetobacter baumannii* CPS

A clinically relevant producer of CPS is the opportunistic pathogen *Acinetobacter baumannii*, which triggers infections in immunocompromised patients causing severe nosocomial, bloodstream, pneumonia, urinary tract infections, and septicaemia [75]. Its clinical importance is related to its low susceptibility towards most of the antibiotics commonly used [76].

There are seven different capsule loci—KL1, KL2, KL4 KL6, KL7, KL8, KL9—in *A. baumannii* genomes. Five of these were found in clonal group 2, whereas two were found in clonal group 1, indicating that isolates with developing antibiotic resistance have a lot of variations of these loci [77]. The K4 CPS of isolate D78, which is a multiple antibiotic resistant strain, contains the KL4 cluster. The KL4 CPS backbone repeating structure is composed of a trisaccharide of α-*N*-acetyl-d-quinovosamine (d-Qui*p*NAc), α-*N*-acetyl-d-galactosamine uronic acid (α-d-Gal*p*NAcA), and α-d-Gal*p*NAc, which contains a branching 4,6-pyruvylated GalNAc residue. The trisaccharide structure was elucidated as →4)[4,6Pyr-α-d-Gal*p*NAc-(1→6)]-α-d-Gal*p*NAc-(1→4)-α-d-Gal*p*NAcA-(1→3)-α-d-Qui*p*NAc-(1→. The pyruvate ketal is predicted to be transferred by the putative pyruvyltransferase PtrA, however, without biochemical evidence [78].

#### 3.2.3. *Klebsiella* CPSs

Pyrogenic liver abscess-causing *Klebsiella pneumoniae* produces a CPS, which is composed of trisaccharide repeating units with the structure →4)-β-d-Glc-(1→4)-2,3(*S*)Pyr-β-d-GlcA-(1→4)-β-l-Fuc-(1→, in which each glucuronic acid residue is pyruvylated and additional acetylation of the fucose residue occurs at the C2-OH or C3-OH [79]. The CPS induces secretion of tumour necrosis factor and interleukin-6 by macrophages through the Toll-like receptor 4 dependent pathway, which is abandoned when pyruvylation is missing in the trisaccharide. This finding indicates that pyruvylation on glycoconjugates may be relevant for immune system stimulation [80]. Previously, the recognition of pyruvylated CPS from *K. pneumoniae* by IgM antibodies has been described [81].

The structures of several other pyruvylated *Klebsiella* CPS structures have been elucidated, however, without any functional information.

The structure of the CPS from *Klebsiella* serotype K14 was the first report on the rare case of a *Klebsiella* polysaccharide to contain a Gal*f* residue. The repeating hexasaccharide structure was shown to terminate with a glucose residue carrying a 4,6Pyr modification—→4)-β-d-Glc*p*A-(1→3)- β-d-Gal*f*-(1→3)-β-d-Glc*p*-(1→4)[4,6Pyr-β-d-Glc*p*-(1→2)][α-l-Rha(1→3)]β-d-Man*p*-(1→ [82].

Also in the doubly pyruvylated CPS of *Klebsiella* K12, Gal*f* residues are found; its repeating unit has the structure 5,6Pyr-β-d-Gal*f*-(1→4)-β-d-Glc*p*A-(1→3)-β-d-Gal*f*-(1→6)-β-d-Glc*p*-(1→3)-α-l-Rha-(1→3)-α-d-Gal*p*-(1→2)[5,6Pyr-β-d-Gal*f*-(1→4)-β-d-Glc*p*A-(1→3)]-β-d-Gal*f*-(1→6)-β-d-Glc*p*-(1→3)-α-l-Rha-(1→3)-α-d-Gal*p* [83].

The structure of the CPS from *Klebsiella* serotype K70 is composed of linear hexasaccharide repeating units that contain a Pyr group attached to a (1→2)-linked α-l-Rha residue in every second repeating unit. The full structure of the *Klebsiella* K70 CPS is →4)-β-d-Glc*p*A-(1→4)-α-l-Rha*p*-(1→2)-α-l-Rha*p*-(1→2)-α-d-Glc*p*-(1→3)-β-d-Gal*p*-(1→2)-3,4Pyr-α-l-Rhap(1→ [84].

The *Klebsiella* serotype K64 CPS consists of hexasaccharide repeating units, composed of a →4)-α-d-Glc*p*A-(1→3)-α-d-Man*p*-(1→3)-β-d-Gal*p*-(1→4)-α-d-Man*p*-(1→ backbone with a 4,6Pyr-β-d-Gal*p* and a l-Rha residue attached to the (1→4)-linked α-d-Man*p* residue at O-2 and O-3, respectively; the repeating unit further contains one *O*-acetyl substituent [85].

The structure of the CPS from *Klebsiella* type K46 consists of a hexasaccharide repeating unit, which is unique in having a 4,6Pyr residue on a lateral, but non-terminal sugar residue—[β-d-Glc*p*-(1→3)-4,6Pyr-β-d-Man*p*-(1→4)]→3)-α-d-Glc*p*A-(1→3)-α-d-Man*p*-(1→3)-α-d-Gal*p*-(1→ [86].

The *Klebsiella* K33 CPS revealed to be a tetrasaccharide alditol with the structure β-d-Glc*p*-(1→4)[3,4Pyr-β-d-Gal*p*-(1→6)]-β-d-Man*p*-(1→2)-Ery-ol, where Ery-ol is erythritol [87].

Interestingly, there are two human monoclonal macroglobulins, IgM^WEA^ and IgM^MAY^ [88], which show specificity for *Klebsiella* polysaccharides containing 3,4-(K30, K33) and 4,6-(K21, K11) pyruvylated d-Gal in a pH-dependent manner and with differences in co-precipitation in dependence of the number of the CPS repeating units (i.e., CPS length) [81]. Of note, agar, which has an internal 4,6Pyr-Gal residue in its repeating unit, cross-reacts with IgM^WEA^ [81].

*Klebsiella rhinoscleromatis* is a heavily capsulated bacterium that possesses a K3-type capsule. The repeating unit of K3 is a pentasaccharide with the structure →2)-4,6-*S-*Pyr-α-d-Man-(1→4)-α-d-GalA-(1→3)-α-d-Man-(1→2)-α-d-Man-(1→3)-β-d-Gal-(1→ [89]. The *Klebsiella* K3 capsule has been shown to be one of the few *Klebsiella* K types that are able to bind to the eukaryotic mannose receptor [90].

#### 3.2.4. *Bacteroides fragilis* CPS A

*Bacteroides fragilis* is an opportunistic anaerobe, most frequently isolated from intra-abdominal abscesses [7,91,92,93]. Its most prominent CPS—CPS A—is composed of tetrasaccharide repeating units with the structure →4)-α-d-2-*N*-acetylamido-4-amino-galactopyranose (AAdGal*p*)-(1→3)-4,6PyrGal*p*-(1→3)-[β-d-Gal*f*-(1→3)]α-d-Gal*p*NAc-(1→) [94]. CPS A has been shown to have a tremendous effect on the immune system of a mammalian host and to be internalized by antigen-presenting cells [7]. Upon genetic deletion of CPS A, the abscess-inducing capability of the bacterium was drastically reduced [93]. CPS A from *B. fragilis* caught recent interest as a carbohydrate antigen to be used in vaccine formulations instead of conventional cationic proteins such as bovine serum albumin (BSA) and keyhole limpet hemocyanin (KHL) [92].

**Figure 3:**
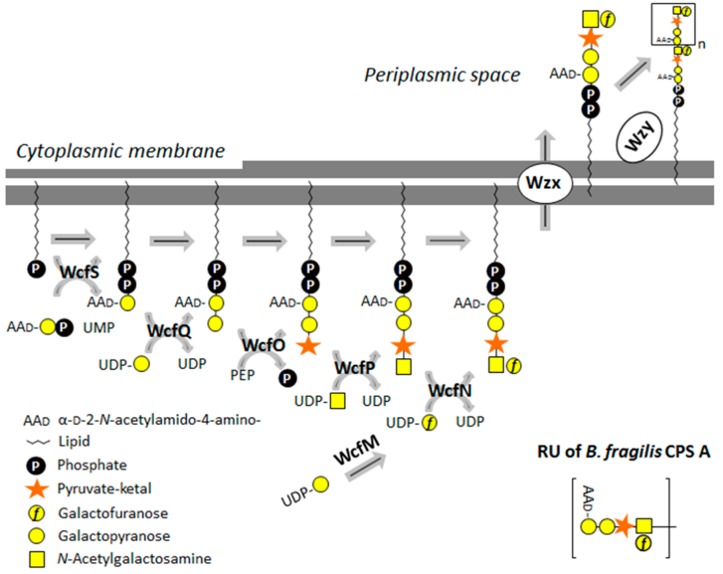
Scheme of capsular polysaccharide (CPS) A biosynthesis in *Bacteroides fragilis*. The pyruvylation step occurs in the cytoplasm at the stage of the lipid-PP-linked RU precursor. Pyruvylation (WcfO) is indicated by a star. Notably, in contrast to succinoglycan biosynthesis (Figure 2), pyruvylation of the internal Gal*p* of the RU needs to proceed prior to completion of the lipid-PP-linked tetrasaccharide repeat. RU: repeating unit. Monosaccharide symbols are shown according to the Symbol Nomenclature for Glycans (SNFG) [45].

CPS A biosynthesis is encoded by a single ~10.7-kb gene locus on the *B. fragilis* genome [93], and predictably employs a Wzx/Wzy-dependent pathway, on the basis of genomic evidence (Figure 3). 

The gene locus encodes four transferases (WcfN, WcfP, WcfQ, and WcfS), where WcfS is responsible for the transfer of the AAdGal*p* residue from its nucleotide activator to an undp-P-lipid carrier as the first step in the synthesis of the CPS A repeating unit, and WcfR is responsible for the prior transfer of the amino group on the AdGal*p* residue to yield AAdGal*p*, which is crucial for virulence. A recent *in vitro* study of the individual enzymatic steps involved in the repeating unit biosynthesis of CPS A yielded first insight into the sugar pyruvylation reaction, with phosphoenolpyruvate (PEP) serving as a donor substrate. There is evidence that pyruvylation occurs on the undp-PP-linked disaccharide repeat unit precursor prior to tetrasaccharide repeat completion, export, and polymerization by a Wzx/Wzy-dependent system [7]. The pyruvyltransferase WcfO from the CPS A biosynthesis of *B. fragilis* [7] is one of the few biochemically characterized enzyme ketal-pyruvyltransferases (for details, see Section 5.1.2).

#### 3.2.5. *Rhodococcus equi* CPS

The bacterial horse pathogen *Rhodococcus equi* elaborates a serotype-specific CPS that functions as a potential virulence factor [94]. This CPS is a high-molecular-weight acidic polymer composed of d-Glc, d-Man, pyruvate, and 5-amino-3,5-dideoxynonulosonic acid (rhodaminic acid, Rho) in the molar ratio of 2:1:1:1. Structural analysis revealed that the CPS consists of linear pyruvylated tetra-saccharide repeats with the structure →3)-β-d-Man*p*-(1→4)-β-d-Glc*p*-(1→4)-α-d-Glc*p*-(1→4)-7,9Pyr-α-RhoAmAc-(2→ [95].

### 3.3. “Non-Classical” Secondary Cell Wall Glycopolymers with Pyruvylated β-d-ManNAc

Anionic secondary cell wall glycopolymers (SCWPs)—among which the wall teichoic acids (WTA) and the lipoteichoic acids (LTA) are best known—serve as a rich source for both validated and unexploited pathways that are essential for bacterial virulence and survival [96,97,98]. Pyruvylated SCPWs are a less-investigated class of peptidoglycan-attached SCWPs that arouse interest because they can be hijacked for a predictably widespread mechanism of protein cell surface display in Gram-positive bacteria [99]. These SCWPs are 5–20 kDa in size, composed of species–specific repeats, but lack repetitive alditol phosphates and phosphodiester bonds typical of WTAs and LTAs [100,101,102]—hence the terminology “non-classical” SCWPs. Importantly, they contain a 4,6-ketal pyruvylated β-d-ManNAc residue (4,6Pyr-β-d-ManNAc), imparting a negative charge and serving as a specific cell wall ligand for S-layer homology (SLH) domains usually present in triplicate at the termini of cell surface proteins [99,103,104]. Among such proteins are S-layer proteins, which self-assemble into 2D crystalline arrays on the bacterial cell surface [105,106]; they are important for many biological functions such as maintenance of cell integrity, enzyme display, protection to phagocytosis, and interactions with the host and its immune system [107]. Because of their unique 2D crystallization ability, S-layer proteins are of great interest for drug delivery, biomaterial engineering, and vaccine development [108].

Fifty-four thousand specific hits within the conserved protein domain family SLH (pfam00395), showing up most prevalent in the *Firmicutes*, *Cyanobacteria*, and *Actinobacteria* phyla of bacteria, emphasize the prevalence of this protein domain. Several bacteria synthesizing a suite of SLH proteins contain pyruvate in their cell wall and have a pyruvyltransferase CsaB ortholog [99,109], which indicates a functional coupling of SLH domains and SCWP pyruvylation.

The best-known pyruvylated SCWPs are those from *B. anthracis* and *B. cereus* strains [14,110,111], and from *P. alvei* [15]. The most evident difference between these SCWPs is the presence of 4,6Pyr-β-d-ManNAc exclusively at the terminal repeat in the former SCWPs, while in that of *P. alvei*, the β-d-ManNAc of each repeat is pyruvylated. This might explain the essentiality of the pyruvyltransferase CsaB in the latter organism [109]. It is conceivable to assume that mono- versus poly-pyruvylation of β-d-ManNAc has implications with regard to the biosynthetic pathway of the respective SCWP, especially the mode of activity of the cognate pyruvyltransferase (compare with Section 5.1.1.).

The genetic determinants and assembly reactions of pyruvylated SCWPs are only beginning to be discovered. Pyruvylated SCWPs are peptidoglycan-linked polymers, as are WTAs [97,98], however, they lack experimental evidence of a comparable biosynthetic route.

#### 3.3.1. *Bacillus anthracis* SCWP

The *B. anthracis* SCWP is composed of →4)-β-ManNAc-(1→4)-β-GlcNAc-(1→6)-α-GlcNAc-(1→ trisaccharide repeats [102] with strain-dependent galactosylation occurring at the GlcNAc residues, and are bound to peptidoglycan via a murein linkage unit [14,112]. Notably this SCWP contains a modified terminal repeat with the structure 4,6-Pyr-β-ManNAc-(1→4)-[3*O*Ac-β-GlcNAc-(1→6)]β-GalN-(1→4) that is pyruvylated and *O*-acetylated [14].

A scenario for the biosynthesis of the *B. anthracis* SCWP has been proposed [113] on the basis of the bioinformatic prediction of four contributing genomic gene clusters and their genetic manipulation, accompanied with analyses of mutant cells. The S-layer gene cluster [99,112,113,114] encodes, among others, components for pyruvylation (*B. anthracis* CsaB) and *O*-acetylation of the terminal SCWP trisaccharide, and a Wzy-like protein. The other gene clusters play predicted roles in the formation of lipid-linked precursors of the murein linkage unit and the trisaccharide repeat [112,114,115,116]. The SCWP biosynthesis model proposes the separate assembly of different undp-PP-linked building blocks in the cytoplasm—the murein linkage unit, trisaccharide repeat, and terminal modified trisaccharide [113]—followed by the individual translocation across the cytoplasmic membrane via Wzx, followed by SCWP polymerization at the outer face of the membrane involving Wzy [113]. This model does not explain how the different building block precursors converge and how pyruvylation of β-d-ManNAc is elaborated. A recent study identified PatB1 as *O*-acetyl-transferase in the terminal repeat biosynthesis of *B. cereus* SCWP, proposing an extracellular *O*-acetylation mechanism [117]. Previously, it was surmised that the modifications at the terminal trisaccharide emerge post-polymerization and ligation to peptidoglycan [14].

*B. anthracis* CsaB is not essential for survival, but it is important for the pathogenesis of infection; Δ*csaB* mutants lacking SCWP pyruvylation fail to retain SLH-domain containing proteins in the cell wall, leading to an atypical cell morphology [99,118]. Of note, in addition to SCWP [14], the *B. anthracis* cell wall contains a polyglycerol phosphate LTA [119] and a poly-γ-d-glutamic acid capsule [101], which could support the cell wall integrity in a strain devoid of pyruvylation providing the anionic character.

#### 3.3.2. *Paenibacillus alvei* SCWP

The SCWP of *P. alvei* consists of →3)-β-d-ManNAc-(1→4)-β-d-GlcNAc-(1→ repeats, where β-d-Man*p*NAc of each disaccharide is modified with 4,6-linked pyruvate ketal [15,120]. Notably, an identical SCWP composition was found in the S-layer carrying *Lysinibacillus sphaericus* CCM 2177, where on average, every second β-d-ManNAc residue contains a 4,6Pyr-modification [121].

*P. alvei* possesses a polycistronic SCWP biosynthesis gene cluster comprised of *csaB*, *tagA, tagO*, two SLH-protein encoding genes—*slhA* and *spaA*—and two ORFs of unknown function [109,122]. For SCWP assembly in *P. alvei*, distinct enzymatic steps have been investigated [109]. The bacterium utilizes the subsequent TagO [123] and TagA [124,125] catalysed reactions—typically involved in WTA biosynthesis to produce the undp-PP-bound murein linkage unit [97,126,127] for the biosynthesis of the lipid-linked disaccharide substrate needed to generate the repeat unit backbone of its SCWP [15]. Pyruvylation with PEP as donor substrate was experimentally determined *in vitro* at the stage of the lipid-linked disaccharide repeat precursor [109] (see Section 5.1.1.).

In *P. alvei*, no viable deletion mutant could be obtained of either *tagO*, *tagA*, or *csaB* [109]—each of which is located on the *P. alvei* genome as a single copy—indicating essentiality of the pyruvylated SCWP for the bacterium. This might be explained by the presence of pyruvylated SCWP as exclusive anionic SCWP in *P. alvei* and may be supportive of the necessity of at least one anionic polymer in the Gram-positive cell wall [128,129].

#### 3.3.3. Cell Wall Polysaccharide of *Paenibacillus polymyxa*

*Paenibacillus* (previously *Bacillus*) *polymyxa* AHU 1385 was among the first bacteria for which a pyruvylated ManNAc residue was described [130]. The pyruvylated epitope is contained in a →3)-4,6Pyr-ManNAc-(1→4)-GlcNAc-(1→-repeating unit of an SCWP that is presumably peptidoglycan-linked. However, its clear that assignment to a specific SCWP class has not yet been reported, nor have any functional implications such as protein binding. Notably, *P. polymyxa* does not possess an S-layer.

### 3.4. SCWPs with Other Pyruvylated Sugar Epitopes

Some SCWPs containing pyruvylated epitopes other than β-d-ManNAc have been reported from the phylum *Actinobacteria*. There, sugar pyruvylation mainly serves as a chemotaxonomic marker of distinct strains, without further knowledge of putative associated functions.

#### 3.4.1. SCWPs from the Genus *Promicromonospora*

Two strains of the genus *Promicromonospora* are recently uncovered examples of bacteria, which possess non-phosphorylated anionic glycopolymers (“non-classical” SCWPs) with pyruvic acid acetals of *R*-configuration in their cell wall [131]. Members of this genus produce a mycelium that fragments into rod-shaped or coccoid elements and are characterised according to different genus-specific chemotaxonomic markers, including the peptidoglycan of the A4α type [132].

The type strain *Promicromonospora citrea* 665^T^ contains two “non-classical” SCWPs, namely a 2-keto-3-deoxy-d-*glycero*-d-*galacto*-nononic acid (Kdn)-teichulosonic acid containing polymer with the repeating unit structure →6)-α-d-Glc*p*(1→6)-α-d-Glc*p*3SO_3_^−^-(1→4)-α-7,9Pyr-Kdn-(2→, where the Kdn residue is 7,9-pyruvylated, and a galactan with the repeating unit structure →3)-α-4,6Pyr-d-Gal*p-*2OAc-(1→, including 4,6-pyruvylation of Gal.

The cell wall of *Promicromonospora* sp. VKM Ac-1028 contains a teichuronic acid-like structure with the repeating unit →6)-α-d-Glc*p*-(1→4)-β-2,3Pyr-d-Glc*p*A-(1→, where glucuronic acid is 2,3-pyruvylated [131].

#### 3.4.2. Teichoic Acids from the Genus *Nocardiopsis*

The first description of a pyruvate ketal modification on a classical teichoic acid (TA) was reported in *Nocardiopsis* strains, a widespread group among the *Actinobacteria* [133]. The genus *Nocardiopsis* is of pharmaceutical and biotechnological interest because of its ability to produce a variety of secondary metabolites—accounting for its wide range of biological activities—and, thus, holds promises as a source of novel bioactive compounds [134].

The major TA of *Nocardiopsis metallicus* VKM Ac-2522T is a 1,5-poly(ribitol phosphate) TA, with each ribitol unit carrying a pyruvate ketal group at positions 2 and 4. The major TA of *N. halotolerans* is a poly(glycerol phosphate-*N*-acetyl-β-galactosaminylglycerol phosphate) structure in which the GalNAc residue carries a 4,6-ketal pyruvate modification.

#### 3.4.3. Teichoic Acids of *Brevibacterium iodinum*

*Brevibacterium iodinum* VKM Ac-2106 produces two distinct WTAs, namely a mannitol-WTA and a glycerol-TA, present in minor amounts. Mannitol-WTA is a 1,6-poly(mannitol phosphate) bearing β-d-Glc*p* residues at the C-2 of mannitol (Man-ol) and, optionally, 4,5-*S*-Pyr residues. Glycerol-WTA is a 1,3-poly(glycerol phosphate) substituted at the C-2 of glycerol by α-d-Gal*p*NAc residues bearing 4,6-*R*-Pyr [135].

### 3.5. Lipopolysaccharides and Lipooligosaccharides

Lipopolysaccharides (LPSs) of Gram-negative bacteria are a unique family of glycolipids based on a highly conserved lipid moiety known as lipid A. These molecules are produced by most Gram-negative bacteria, in which they play important roles in the integrity of the outer-membrane permeability barrier and participate extensively in the host–pathogen interplay [136,137]. Complete LPSs have a three-domain molecule architecture; the two-domain variants without an O-antigenic polysaccharide (O-PS) are termed lipooligosaccharides (LOSs) [138]. Lipid A is the hydrophobic anchor of LPSs; it is a unique phosphoglycolipid containing glucosamine (GlcN) residues, which are present as β-(1→6)-linked dimers. The disaccharide contains phosphoryl groups and (*R*)-3-hydroxy fatty acids in ester and amide linkages. Variations in the fine structure can arise from the type of hexosamine present, the degree of phosphorylation, the presence of phosphate substituents, and, importantly, in the nature, chain length, number, and position of the acyl groups. Lipid A is glycosylated with a core oligosaccharide (core OS)—typically containing Kdo, a signature molecule of LPS [139], and heptose residues, which may provide an attachment site for a long-chain O-PS of varying repeating unit composition. The O-PS provides a major cellular antigen (O-antigen) used for serological typing of clinical isolates of a given species. Notably, the O-antigen is expressed by most of the clinically relevant strains and is an important phage receptor; LOS, in contrast has been found to be expressed by a group of Gram-negatives that colonize genital and respiratory mucosal surfaces [140].

#### 3.5.1. *Pseudomonas stutzeri*

Analysis of the LPS of *Pseudomonas stutzeri* OX1 revealed a novel type of highly negatively charged LOS, containing two 4,6-linked pyruvate ketals linked to *N*-acetylglucosamine and glucose, independently [4]. The overall LOS structure has been determined to be 4,6(*S*)Pyr-β-Glc-(1→3)[4,6(S)Pyr-β-GlcNAc](1→4)GalNAc(1→3)-Hep7Cm2*P*4*P*-(1→3)-Hep2*P*4*P*-(1→5)[Kdo-(2→4)]Kdo-(2→6)-β-GlcN4*P*-(1→6)-GlcN1*P*, where *P* represents a phosphate group and Cm is carbamoyl. *P. stutzeri* OX1 was isolated from the activated sludge of a wastewater treatment plant, where unusual metabolic capabilities for the degradation of aromatic hydrocarbons were found. Pyruvate residues might be used to block elongation of the LPS chain to yield an LOS. This would lead to a less hydrophilic cellular surface, indicating an adaptive response of *P. stutzeri* OX1 to a hydrocarbon-containing environment [4].

#### 3.5.2. *Providencia alcalifaciens*

Bacteria of the genus *Providencia* are opportunistic human pathogens that cause intestinal and urinary tract infections. The O-antigen-based serological classification scheme of *Providencia alcalifaciens, Providencia rustigianii*, and *Providencia stuartii* includes 63 O-serogroups [141], most of which are acidic. Complex core structures have been elucidated in several *Providencia* O-serogroups [142,143].

*P. alcalifaciens* O19 differs in its O-PS from other *Providencia* strains. The O-PS repeat contains a 4,6-pyruvylated GlcNAc residue and has the complete structure →2)-β-Fuc3NAc4Ac-(1→3)-4,6(*S*)Pyr-α-GlcNAc-(1→4)-α-Gal-(1→4)-β-Gal-(1→3)-β-GlcNAc-(1→. In the NMR spectra of the oligosaccharide, signals of the methyl group of pyruvic acid have been observed and the presence of pyruvic acid in two thirds of the O-units in the polysaccharide has been proven [141].

#### 3.5.3. *Shigella dysenteriae*

*Shigella dysenteriae* is an aetiological agent of various intestinal disorders, including shigellosis. The strains of this bacterium are serologically heterogeneous because of the diversity of the structures of their O-antigens [144].

The structure of the *S. dysenteriae* type 10 O-antigen has been revised by Perepelov et al. in order to account for the acid-labile pyruvate modification that has been lost in a previous investigation due to acidic treatment of the sample. The full O-PS structure has been elucidated to be →2)-4,6(*S*)Pyr-β-d-Man*p*-(1→3)-α-d-Man*p*NAc-(1→3)-β-l-Rha*p*-(1→4)-α-d-Glc*p*NAc-(1→ [145].

#### 3.5.4. *Raoultella terrigena*

The enterobacterium *Raoultella terrigena* is another bacterium that carries a pyruvic acid modification on its O-PS β-Man residue, located at the O-4 and O-6 positions [146]. The structure of the repeating unit of the O-PS has been determined by means of chemical and spectroscopic methods and found to be a linear tetrasaccharide with the structure →2)-4,6(*S*)-Pyr-β-d-Man*p*-(1→3)-α-d-Man*p*NAc-(1→3)-β-l-Rha*p*-(1→4)-α-d-Glc*p*NAc-(1→ [146], which is identical to that of *S. dysenteriae* type 10 [145].

#### 3.5.5. *Proteus mirabilis*

The O-PS repeating unit of *Proteus mirabilis* O16 has been established to be →3)-β-d-Glc*p*NAc-(1→3)-4,6-*R*-Pyr-α-d-Gal*p*NAc1→4)-α-d-Gal*p*A-(1→3)-α-l-Rha*p*2Ac-(1→ [147]. This structure is significantly different from the O-PS structures of other *Proteus* spp. from the serogroup O19, such as *Proteus vulgaris, Proteus hauseri*, and *Proteus penneri* strains, and thus was a key for the reclassification of various *Proteus* strains.

#### 3.5.6. *Cobetia pacifica*

The O-PS form the LPS of *Cobetia pacifica* KMM 3878—an aquatic isolate form Japan—is composed of sulphated and pyruvylated trisaccharide repeats with the structure →4)-β-d-Gal-2,3-SO_3_H-(1→6)-β-d-Gal-3,4-*S*-Pyr-(1→6)-β-d-Gal-(1→ [148].

### 3.6. Mycobacterial Glycolipids with Pyruvate

Mycobacteria contain a variety of glycolipids, including, among others, acylated glucose, acylated trehalose, sulfatides, mannophosphoinositides, and glycopeptidolipids [149]. A crude glycolipid fraction from *Mycobacterium smegmatis* ATCC 356 obtained by ethanolic extraction and silica gel chromatography revealed the presence of hitherto unknown anionic glycolipids [150]. The corresponding glycan moiety has the structure 4,6Pyr-3-*O*-Me-β-d-Glc*p*-(1→3)-4,6Pyr-β-d-Glc*p*-(1→4)-β-d-Glc*p*-(1→6)-β-d-Glc*p*-(1→1)-α-d-Glc.

Members of the *Mycobacterium avium–Mycobacterium intracellulare* (MAI) complex are typeable on the basis of their specific antigenic glycolipid. For instance, the dominant epitope of the MAI serovar 8-specific glycopeptidolipid is a terminal 4,6Pyr-*O*-Me-α-d-Glc*p* unit, whereas that of the MAI serovar 21 has the same terminal pyruvylated glucose devoid of the 3-methoxy group [151]. Healthy individuals of some populations are carriers of antibodies that are specific to these pyruvylated epitopes on the glycopeptidolipids. It is currently unclear, if the antibody reflects previous experience with one or both of these serovars or whether some other common cross-reacting pyruvylated environmental antigen is involved [151]. However, this finding might have protective implications against mycobacterioses and other infectious diseases.

### 3.7. Pyruvylated Glycoconjugates in Eukaryotes

#### 3.7.1. Eukaryotic Glycolipids

Information on pyruvylated glycoconjugates in eukaryotes is scarce in comparison to their description in bacteria. It is currently not clear whether this reflects the natural distribution of pyruvylation or if pyruvylation on eukaryotic glycoconjugates has escaped detection. Notably, pyruvylation has so far not been detected in humans.

An “exotic” example of a pyruvylated eukaryotic glycoconjugate is the phosphonoglyco-sphingolipid containing pyruvylated galactose in the nerve fibres of the sea hare *Aplysia kurodai*. The glycan structure of this phosphonoglycosphingolipid is 3,4Pyr-β-Gal-(1→3)-α-GalNAc-(1→3)-α-Fuc-(1→)-2-aminoethylphosphonyl-Fuc-(1→6)-β-Gal-(1→4)-Glc-(l→ [152].

#### 3.7.2. *N*-Linked Glycans in Yeast

Yeast species are known for the production of high- or oligo-mannosidic *N*-glycans that are displayed on various cell surface proteins [153]. In several yeast species (e.g., *Saccharomyces cerevisiae*, *Candida albicans*, *Pichia holstii*, and *Pichia pastoris*), phosphate groups or, to a lesser extent, sialic acids present on these extracellular glycans provide the necessary negative cell surface charge [153,154,155].

*S. pombe* is a notable example of a yeast whose net negative surface charge is neither conferred by phosphate nor by sialic acid. Instead, the *N*-linked galactomannans of *S. pombe* have pyruvylated β-Gal-(1→3)-(PvGal) caps on a portion of the α-Gal-(1→2)-residues in their outer *N*-glycan chains [156]. *S. pombe* lacks the ER Man_9_-α-mannosidase function as known from, for example, *Saccharomyces cerevisiae*. Therefore, it adds further mannose and galactose residues to the common *N*-glycan core structures, yielding galactomannans [157,158,159]. At least five different genes are required to synthesize the PvGal epitope. It is assumed that 4,6Pyr-β-Gal-(1→3) synthesis is carried out by a coordinated enzymatic system in which the β-Gal-(1→3) residues are first added to the *S. pombe* galactomannans and subsequently pyruvylated by the pyruvyltransferase Pvg1p [3] (see Section 5.1.3.). However, the complete mechanism for PvGal biosynthesis is currently unknown [3].

4,6Pyr-β-Gal is predicted to be the only contributor to the net negative cell surface charge of yeast, as disruption of the *pvg1+* gene resulted in charge abolishment [160].

#### 3.7.3. Pyruvylated Galactans of Algae

Pyruvylated galactan sulphates are often found in red algal polysaccharides, which generally contain 3-substituted 4,6Pyr-d-Gal*p* residues. Among these galactans is that of *Palisada flagellifera*, which represents a highly complex structure with at least 18 different types of derivatives that are found mostly pyruvylated, 2-sulfated, and 6-methylated [161]. Another galactan is that of *Solieria chordalis*, the structure of which remains unknown but was shown to have high immunostimulating potential [162]. Other examples include the carragenans from Australian red algae of the family *Solieriaceae* [10] and galactans of the red seaweed *Cryptonemia crenulata* [13].

Examples of green algae include the highly pyruvylated and sulfated galactans from tropical green seaweeds of the order *Bryopsidalesor*, which have anticoagulant activity [11], such as that of *Codium divaricatum* with the structure Gal*p*-(4SO_4_)-(1→3)-Gal*p*-(1→3)-Gal*p*-(1→3)-Gal*p* and 3,4Pyr-Gal*p*-(6SO_4_)-(1→3)-Gal*p* [12].

#### 3.7.4. Pyruvylated Proteoglycan

The marine sponge *Microcionia prolifera* produces a pyruvylated adhesion proteoglycan with the structure 4,6Pyr-β-Gal-(l→4)-β-GlcNAc-(l→3)-Fuc-(1→ that is involved in species-specific cell re-aggregation [163].

## 4. Methods for Research of Pyruvylated Glycoconjugates

### 4.1. Isolation of Pyruvylated Bacterial Glycoconjugates

Several protocols for the isolation of glycoconjugates are in use; however, there is no specific general protocol for pyruvylated glycoconjugates. The procedures are strongly dependent on the source of the glycoconjugate—with a special emphasis on the cell wall architecture (i.e., Gram-positive versus Gram–negative bacteria)—and the class of glycoconjugate. Further, for each studied organism, the extraction protocol needs to be optimised. Because of the chemical nature of the acid-labile pyruvate entity, as the only commonality, for the isolation of pyruvylated glycoconjugates, acidic conditions should be avoided to prevent the loss of pyruvate [9,100,145].

For the extraction of EPS, for instance, the types of interactions by which the EPS matrix is created need to be taken into account, including variable extents of electrostatic interactions, van der Waal forces, hydrogen bonds, and hydrophobic interactions [164]. In most cases, physical forces are used to extract EPSs, such as centrifugation and filtration [28], stirring, pumping or shaking, heat treatment, or sonication [164]. Chemical steps include alkaline treatment with NaOH, addition of EDTA for removal of cations, addition of NaCl, use of ion exchange resins (e.g., Dowex), or enzymatic treatment [63,165]. If proteases are used for break-down of co-isolated proteins, an *O*-deacetylation step needs to be introduced to avoid the loss of putative acetyl groups on the EPS [55]. All mentioned chemical additives increase the solubility of the EPS in the aqueous phase; to solubilise EPS with hydrophobic portions, such as that from *Klebsiella pneumoniae*, detergents are necessary [166]. For the precipitation of EPS from the aqueous phase, ethanol is routinely used [65]. To enhance the EPS yield, often a combination of physical and chemical methods is applied [164].

For the extraction of CPS from Gram-negative bacteria, again, NaCl and EDTA are recommended [167]. Other protocols for the release of CPS are based on heat treatment of cells followed by precipitation of the CPS with acetone [80,90].

The isolation of SCWPs—classical and “non-classical” forms—is divided in two main steps: the purification of the peptidoglycan sacculus, which includes treatment of cells with heat, SDS, nuclease, and a protease such as trypsin, and extraction of SCWP by either ethanol precipitation for WTAs, or hydrofluoric acid treatment followed by ethanol precipitation for “non-classical” SCWPs [115,168,169]. The isolation of “non-classical” pyruvylated SCWP of *B. anthracis* was recently described in detail [169].

The extraction of LPS and other cell surface polysaccharides has been described previously [170,171]. Prior to extraction of LPS, pelleted Gram-negative bacteria are usually depleted from CPS by aqueous washing [171]; most commonly, LPS is extracted [146,172,173] or, in the case of LOS, with phenol/chloroform/petrol ether (PCP) [174,175]. The crude extracts are subsequently de-*O*/*N*-acylated under mild acidic or basic conditions, with a preference for the latter. Further purification of the samples can be achieved by size exclusion and/or ion exchange chromatography [176].

### 4.2. Pyruvate Analytics

#### 4.2.1. Lectin Approach

Serum amyloid P component (SAP)—a normal plasma glycoprotein—has a Ca^2+^-dependent binding specificity for 4,6Pyr-*O*Me-β-d-Gal*p* (MOPDG) [177], and thus behaves like a lectin and may be a useful probe for this epitope as present in the cell walls of bacteria and other organisms [178]. SAP has been found to bind *in vitro* to *K. rhinoscleromatis* [89], the cell wall of which is known to contain this particular pyruvylated epitope. Binding was shown to be less pronounced to *X. campestris*, which contains a 4,6Pyr-Man*p* epitope [18], and no SAP bound to *E. coli*, which contains pyruvate 4,6-linked to glucose or to *S. pneumoniae* type 4, which contains pyruvate 2,3-linked to Gal*p* [74]. Binding of SAP to those organisms, which it did recognise, was completely inhibited or reversed by millimolar concentrations of free MOPDG.

#### 4.2.2. Biochemical Pyruvate Assays

A specifically developed colorimetric/fluorometric assay for ketal-pyruvate detection via enzymatic oxidation has been incorporated in a recently introduced high throughput screening platform for the structural analysis of novel EPS structures [179], which underlines the importance of pyruvylated epitopes. The platform is based on ultra-high performance liquid chromatography coupled with ultra-violet and electrospray ionization ion trap detection following EPS isolation.

A similar procedure for detection of free pyruvate is used in clinics. Pyruvate serves as an important metabolite in the citric acid cycle for the screening of liver diseases and genetic disorders in humans, as these are reflected by high pyruvate levels [180]. The procedure is based on the oxidation of pyruvate by pyruvate oxidase in the presence of acetyl phosphate, which leads to the production of CO_2_ and H_2_O_2_. The latter is detected via a fluorometric probe followed by a horseradish peroxidase reaction, which leads to the formation of resorufin. Colour development can be detected at 570 nm, and fluorescence at 530–540 nm for excitation and 585–595 nm for emission (Cayman pyruvate assay kit: https://www.caymanchem.com/pdfs/700470.pdf).

Other methods for pyruvate detection stem from food analytics, as pyruvate is involved in the degree of pungency of onions. Different methods are on the basis of the determination of total 2,4-dinitrophenylhydrazine-reacting carbonyls in a sample by photometric detection. Furthermore, oxidation of reduced diphosphopyridine nucleotide (DPNH) by pyruvate can be measured in a coupled reaction with lactic dehydrogenase. Decrease of the absorbance at 340 nm correlates with the oxidation of DPNH and, therefore, the concentration of pyruvate [181,182].

Assaying pyruvylation reactions of monosaccharides using HPLC-based approaches is dependent on the intended mode of detection. Frequently, specifically introduced saccharide modifications are used for detection purposes. One prominent example is the chemical attachment of *para*-nitrophenol (pNP) to the saccharides of interest for monitoring at 265 nm. To determine, for instance, the activity of the yeast pyruvyltransferase Pvg1p, the pyruvylated product species was separated from the unpyruvylated educt species using a COSMOSIL 5C18-P revered phase (RP) C18 column with 0.3% ammonium acetate, pH 7.4, containing 13% acetonitrile as a solvent. The pyruvylated product eluted from the column earlier than the educt, as monitored by recording the absorbance at 265 nm [160]. Another option is the use of a RP-C18 column in combination with a 1-propanol gradient in 88% 100 mM ammonium bicarbonate, accompanied by the detection of the nitrophenyl-modified sugar at an absorbance of 405 nm [7].

A more sophisticated fluorescent polyisoprenoid chemical probe—2-amideaniline-undP-PP-AAdGal-Gal, which equals an acceptor substrate mimic from the *B. fragilis* CPS A tetrasaccharide biosynthesis pathway—was established by Sharma et al. to monitor pyruvylation of the fluorescent lipid-linked substrate by the pyruvyltransferase WcfO directly by HPLC on a C18 column. An isocratic gradient of 35% 1-propanol with 65% 100 mM ammonium bicarbonate was used, and detection was done by fluorescence at excitation at 340 nm, and emission at 390 nm [7].

#### 4.2.3. NMR Analysis of Pyruvylation

**Figure 4:**
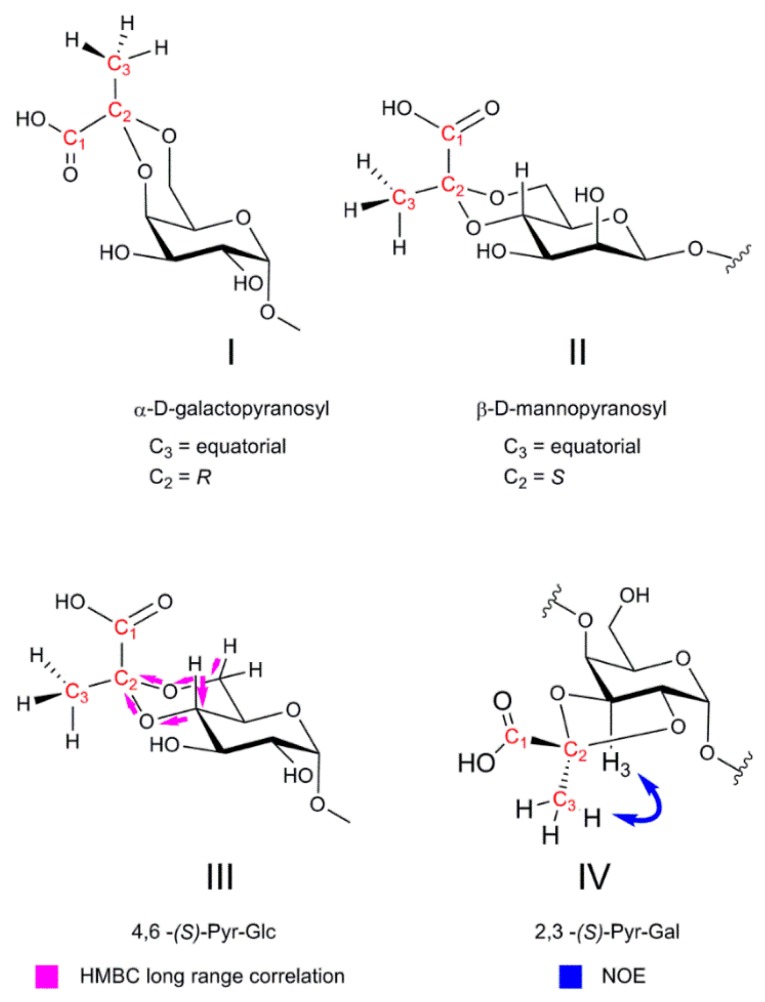
**I** and **II**, equatorial-oriented methyl groups of 4,6 *galacto*- and *manno*-pyranosyls. **III**, identification of the attachment site of pyruvylation via hetero multiple bond correlation (HMBC). **IV**, through-space correlation of the pyruvate methyl group to a ring proton—in this case H_3_. Pink arrows indicate though-bond interactions of neighbouring protons (HMBC), while blue arrows indicate through-space interactions of neighbouring protons (NOE).

Nuclear magnetic resonance (NMR) is a versatile tool for the non-invasive structure elucidation of bacterial polysaccharides, including substitutions such as pyruvic acid [183], which can be frequently found as 4,6-*O*, 3,4-*O*, or 2,3-*O* acetals. Systematic investigations of defined pyruvylated monosaccharides revealed stereospecific repeating patterns from which the absolute configuration of pyruvic acid acetals can be inferred [184]. It was shown that the ^13^C signal of an equatorial 4,6-pyruvate methyl group (Figure 4I,II) resonates at ~26 ppm, while the axial methyl group can be found at ~17 ppm. For 3,4 acetals, the ^13^C shifts have been studied in detail, and the difference between axial and equatorial methyl groups was found to be much smaller in comparison to 4,6. The ^1^H difference, however, is in this case more noticeable [185]. The ring form of the acetal being either 5- (for 3,4-O) or 6- (for 4,6-O) membered is reflected by ^13^C shifts [186]. It has also been shown that for most 4,6 acetals, the configuration of the methyl group is equatorial, which results in an *S* configuration for the d-*gluco-* and d-*manno*-pyranosyls and an *R* configuration for the d-*galacto*-pyranosyls [187].

In ^1^H NMR, the presence of pyruvic acid (4,6-, 3,4-, 2,3-) is usually indicated by a single prominent signal of the methyl group between 1.3–1.7 ppm, with a threefold higher relative intensity (peak area) in relation to another indicative signal such as the anomeric proton. For repeating units of polymers, the peak area of the methyl signal relative to another indicative signal reveals the degree of pyruvate substitution.

In ^13^C NMR, the presence of pyruvate substitution is usually indicated by the presence of signals for the pyruvic methyl group around 17–30 ppm (Figure 4, C_3_). The quaternary acetal carbon (Figure 4, C_2_) resonates in the anomeric region around 100 ppm (for 4,6-*O*) or 110 ppm (for 3,4-*O* and 2,3-*O*). Additionally, the quaternary signal of the carboxylic acid (Figure 4, C_1_) can be found between 170 and 180 ppm, with the 4,6-pyruvates present more towards 170 ppm and the 2,3 and 3,4 acetals found closer to 180 ppm [131].

The connectivity between the pyruvate and a saccharide is routinely determined by the employment of long-range ^1^H-^13^C correlation detection methods such as hetero multiple bond correlation (HMBC) experiments, which usually give correlation information over three and more bonds from the corresponding ring protons to the quaternary carbon of the acetal (Figure 4III). Therefore, 4,6-, 3,4-, or 2,3-pyruvic acetal identification is straightforward. The absolute configuration of the pyruvic acid acetal can be confirmed by through-space correlation experiments such as 1D or 2D NOESY (nuclear Overhauser and exchange spectroscopy), ROESY (rotating frame Overhauser enhancement spectroscopy), or GOESY (gradient nuclear Overhauser and exchange spectroscopy) (Figure 4, IV) [74,146,188,189].

#### 4.2.4. MS analysis of Pyruvylation

Mass spectrometry in combination with NMR is a very powerful tool to determine the presence and position of pyruvylation in oligo- or polysaccharides. Common approaches are based on the break-up of polysaccharides by acid hydrolysis, methanolysis, and then either silylation [59,66] or acetylation [148], followed by gas chromatography (GC) or electrospray ionization-mass spectrometry (ESI-MS) analysis of the resulting monosaccharides [190]. Usually, characteristic patterns in the mass spectrum at the monosaccharide level are observed in the presence of pyruvates, such as a characteristic fragment ion at *m/z* 363 (M-COOMe) consistent with a molecular mass of 422, which would be indicative of a methyl *O*-(1-carboxyethylidene)hexopyranoside methyl ester di-O-trimethylsilyl-ether. Comparison of the methylation analysis on native and depyruvylated polysaccharides allows for the pinning down the initial position of the acetalic linkages. Methanolysis and reductive cleavage have been described for the analysis of pyruvate-containing polysaccharides [191]. General procedures for the MS analysis of oligosaccharides have been reviewed in detail elsewhere [192,193].

## 5. Ketal-Pyruvyltransferases

### 5.1. Substrate Specificity of Ketal-Pyruvyltransferases

The pyruvyltransferase CsaB from the SCWP biosynthesis pathways of *P. alvei* [109], the pyruvyltransferase WcfO from CPS A biosynthesis of *B. fragilis* [7], and the pyruvyltransferase Pvg1p from the *N*-glycan biosynthesis of *S. pombe* [161] are among the few studied enzyme orthologues.

#### 5.1.1. CsaB from *P. alvei*

*P. alvei* CsaB catalyses the pyruvate modification on a β-d-ManNAc residue present in every SCWP repeat; the resulting 4,6-β-d-ManNAc is the essential epitope for the binding of the bacterium’s SLH domain-containing S-layer protein SpaA, as revealed from the co-crystal structure of synthetic pyruvylated ligand with truncated SpaA_SLH_ [104,194]. Supporting data comes from isothermal titration calorimetry, revealing binding between these modules only when the pyruvate entity was present [104].

Notably, a comparable mode of binding is elaborated between the S-layer protein Sap of *B. anthracis* and its pyruvylated SCWP [195]. However, poly-pyruvylation, as in the *P. alvei* SCWP, is not found in the *B. anthracis* SCWP, where only the β-d-ManNAc of the terminal repeat is modified [14].

For *B. anthracis*, a model for a Wzx/Wzy-dependent biosynthesis has been proposed, including cytoplasmic pyruvylation of the terminal repeating unit; however, the model is without any evidence of the nature of the acceptor substrate and biochemical proof of CsaB activity [113].

**Figure 5:**
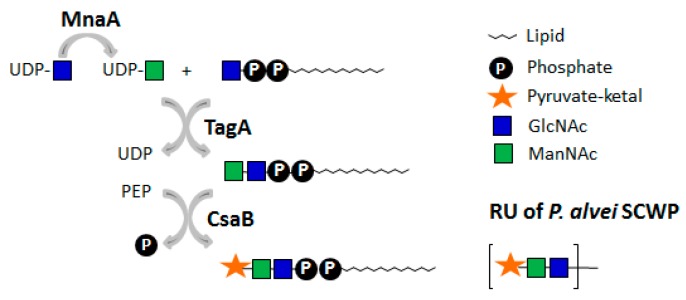
*In vitro* one-pot reaction involving the *Paenibacillus alvei* enzymes MnaA, TagA, and CsaB in combination with a synthetic lipid-PP-GlcNAc primer, demonstrating the preference of *P. alvei* CsaB for a lipid-PP-linked disaccharide substrate [109]. Pyruvylation (CsaB) is indicated by a star. RU: repeating unit. Monosaccharide symbols are shown according to the Symbol Nomenclature for Glycans (SNFG) [45].

Concerning the pyruvyltransferase CsaB from *P. alvei*, in an *in vitro* enzyme assay, the necessity of a lipid-portion of the acceptor could be unambiguously demonstrated; neither free ManNAc nor a nucleoside-diphosphate-linked substrate was accepted as a substrate [109]. Using *P. alvei* TagA in combination with a synthetic 11-phenoxy-undecyl-PP-α-GlcNAc acceptor and UDP-ManNAc as substrate, it was demonstrated that TagA is an inverting UDP-α-d-ManNAc:GlcNAc-lipid carrier transferase of *P. alvei*. The produced 11-phenoxyundecyl-PP-α-d-GlcNAc-(1→4)-β-d-ManNAc compound was an acceptor substrate for 4,6-ketalpyruvyl transfer catalysed by recombinant *P. alvei* CsaB using PEP as a donor substrate [109], yielding a lipid-PP-linked pyruvylated disaccharide precursor (Figure 5).

Subsequent steps of SCWP biosynthesis in *P. alvei* remain elusive, as there is currently neither in silico nor experimental data available favouring a Wzx/Wzy- or ABC transporter-dependent pathway.

#### 5.1.2. WcfO from *B. fragilis*

The necessity of a lipid-PP-bound substrate for pyruvyltransferase activity is supported by studies on the pyruvyltransferase WcfO from the *B. fragilis* CPS A biosynthesis; CPS A is composed of tetrasaccharide repeats containing an internal 4,6Pyr-Gal residue. On the basis of the stepwise enzymatic processing of an undp-PP-AAdGal*p* precursor *in vitro*, pyruvylation by WcfO was predicted to occur in the cytoplasm at the stage of the lipid-linked CPS A repeat unit precursor undp-PP-AAdGal*p*-Gal before completion of the tetrasaccharide repeat and completion of the CPS A in the periplasm [7,93] (Figure 3). Importantly, WcfO was inactive on UDP-galactose or pNP-galactose, supporting the requirement of a lipid-P carrier for pyruvyltransferase activity of WcfO [7].

Enzymatic transfer of pyruvate onto lipid-bound sugar intermediates has also been previously described in CPS biosynthesis of *Rhizobium trifolii* [196,197] and in xanthan biosynthesis of *Xanthomonas campestris* [31].

#### 5.1.3. Pvg1p from *S. pombe*

In contrast, the third functionally characterized 4,6-ketal-pyruvyltransferase, Pvg1p from *S. pombe*, was proven *in vitro* with both pNP-β-Gal and pNP-β-lactose serving as suitable acceptor substrates [160]. According to studies of the pyruvylation mechanism, Pvg1p resides in the membrane of the Golgi apparatus where it adds the pyruvate moiety to the Gal caps of its *N*-glycans. For this purpose, PEP is transported by two transporters, Pet1p and Pet2p, into the lumen of the Golgi apparatus where it serves as a donor substrate for the pyruvylation reaction [160].

A recent study has determined the crystal structure of the Pvg1p enzyme [160]. Pvg1p consists of 12 α-helices and 12 β-sheets, with 2 α/β/α domains at the N- and C-terminal half regions. Charged surface representation analysis revealed a positively charged cleft situated between the N- and C-terminal halves of Pvg1p, which suggests a possible mode of binding that may accommodate the negatively charged PEP donor substrate. Since neither PEP- nor pNP-β-Gal-co-crystal structures with the enzyme could be obtained, the empty substrate-binding cleft was used as a scaffold for computational substrate modelling using PEP [198]. In the proposed computational model, residues R217, R337, L338, and H339 form direct hydrogen bond contacts with PEP. Residues L338, H339, and D240 also appear to function in maintaining the shape of the PEP-binding pocket via a set of specific interactions. The crystallization study indicated that the pyruvylation process mimics sialyation; interestingly, Pvg1p shows resistance to sialidase digestion. Thus, a better characterization of the effects of pyruvylation might facilitate the development of pharmaceutical glycoproteins [198]. From the same research group, an enzyme was characterised as a 4,6Pyr-β-d-Gal-releasing enzyme (PyrGal-ase) with specificity for the (1→3) yeast linkage; mammalian (1→4)-linked PyrGal could not be hydrolysed. The physiological role of the PyrGal-ase in the *Bacillus* strain from where it was isolated is currently unknown [199].

Except for the three characterized pyruvyltransferases, no data on neither the activity nor the substrate specificity of pyruvyltransferases is available in the literature. This is surprising, considering that pyruvylation on glycoconjugates is widely distributed in nature. Future research on ketal-pyruvyltransferases should be directed towards mechanistic investigations of the enzyme’s mode of catalysis, as well as inhibitor screening, similar to that of the enol-pyruvyltransferase MurA, which is a prominent target of antibiotics.

### 5.2. Challenges in Research of Ketal-Pyruvyltransferases

Currently, no definite classification of pyruvyltransferases is possible, although orthologous enzymes are predicted in various organisms. The Carbohydrate-Active enZYme (CAZy) database (http://www.cazy.org/) reveals, for instance, putative polysaccharide pyruvyltransferases from *Clostridium stercorarium* subsp. *stercorarium* DSM 8532 and *Clostridium thermosuccinogenes* DSM 5807 belonging to the glycosyltransferase 4 (GT4) family, with a classification as retaining GT type B fold-like glycosyltransferases.

The reasons for the limited number of characterised pyruvyltransferases are due to the challenges faced with the set-up of *in vitro* enzyme assays. While commercially available PEP has been proven to be a suitable donor substrate for the transfer of the pyruvyl moiety in distinct cases [7,109,195], the availability of suitable acceptor substrates is a limiting factor. Free saccharides have not been recognised as acceptor substrates by the pyruvyltransferases investigated so far [7,109]. According to our current knowledge, these enzymes instead require more elaborate intermediates from the pyruvylated glycoconjugate’s biosynthesis pathway. Depending on the glycoconjugate structure and its mode of biosynthesis—which might be an *en bloc* (involving an ABC transporter) or sequential synthesis (involving a Wzx flippase and a Wzy polymerase) according to the terminology introduced for LPS biosynthesis routes [97,98,138,200], yielding pyruvylation as either a pre- or post-polymerization modification—di-, tri-, or even oligosaccharide repeating units might be required. Furthermore, most glycoconjugates are biosynthesized on a membrane-embedded lipid carrier, such as undp-P or diacylglycerol. Such lipid-linked glycan precursors usually cannot be purified from the natural source in sufficient quantity and purity because of the high turnover rates and efficient recycling pathways of these lipid carriers, which are shared between several cellular glycoconjugate biosynthesis pathways, including that of peptidoglycan [201].

Thus, complex saccharide acceptor substrates are required, which are not commercially available. These compounds need to be produced along sophisticated and laborious chemical synthesis schemes, which also need to account for a lipophilic portion, either in the form of the native lipid carrier or a simplified mimic thereof.

For identifying a suitable acceptor substrate for an *in vitro* pyruvyltransferase assay, a delicate balance between the best possible acceptor mimic and solubility needs to be found in order to enable subsequent analytical procedures. To overcome all these challenges, the development of novel chemical, enzymatic, or chemo-enzymatic synthesis strategies for acceptor substrate production is a current major focus in pyruvyltransferase research.

### 5.3. Sequence Space of Ketal-Pyruvyltransferases

This review aimed at exploring the currently known sequence variation (extant sequence space) of pyruvyltransferases and their taxonomic distribution on the basis of the three functionally characterized sequences—*P. alvei* CsaB (K4ZGN3), *S. pombe* Pvg1p (Q9UT27), and *B. fragilis* WcfO (Q5LFK7).

#### 5.3.1. Methods

The best 50 sequence hits of BLAST searches with K4ZGN3_CsaB, Q9UT27_Pvg1p, and Q5LFK7_WcfO were aligned with MAFFT using the algorithm FFT-NS-2. The three alignments were then used as queries for hmmsearch [202] on the UniProtKB database, setting significant E-values for sequences <9.0 × 10^−30^ and for hits <9.9 × 10^−30^. Results were restricted to hits showing a pyruvyltransferase domain (PS_pyruv_trans domain; Pfam: PF04230). The resulting three sequence selections were filtered for incomplete sequences and annotated according to their taxonomy using the online tool SeqScrub [203]. The sequences were further submitted to the Enzyme Function Initiative-Enzyme Similarity Tool (EFI-EST) [204] with an initial BLAST E-value of 1 × 10^−5^ to calculate sequence similarity networks (SSNs). Sequences were restricted to a length between 250 and 600 amino acids, and the calculated networks were displayed at an alignment score cut-off of 1 × 10^−50^.

#### 5.3.2. Results

Three independent database searches based on the biochemically characterized pyruvyltransferases CsaB, Pvg1p, and WcfO resulted in three sequence selections of 2053, 1019, and 233 sequences, respectively. When comparing these selections, it was found that they did not share any protein sequences, implicating that the sequence space covered by these searches does not overlap. It is, therefore, conceivable to assume that more pyruvyltransferase sequences and organisms harbouring a pyruvyltransferase gene exist, which are not covered in this study. Additionally, the comparison shows that there are at least three different types of pyruvyltransferases that do not share a close sequence relationship. Judging from the number of sequences in the selections and the extent of their taxonomic distribution, pyruvyltransferases from the SSN of CsaB (CsaB-like), and pyruvyltransferases from the SSN of Pvg1P (Pvg1P-like) seem to be the most common types of pyruvyltransferases, while WcfO-like pyruvyltransferases might be more of a specialized type of pyruvyltransferase.

**Figure 6:**
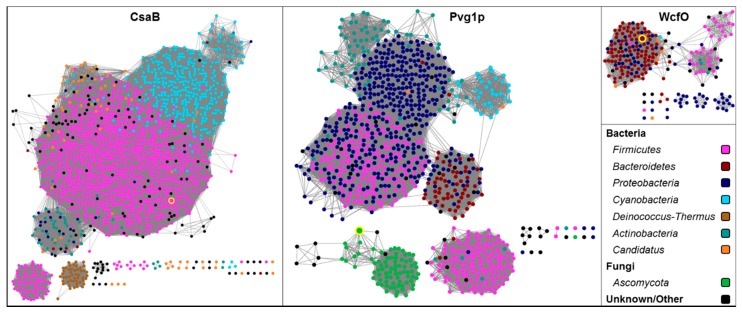
Sequence similarity networks illustrating the extant sequence space around *Paenibacillus alvei* CsaB (K4ZGN3), *Schizosaccharomyces pombe* Pvg1p (Q9UT27), and *Bacteroides fragilis* WcfO (Q5LFK7). The three characterized sequences—CsaB, Pvg1p, and WcfO—are highlighted by yellow circles.

Looking at the taxonomic distribution of these types of pyruvyltransferases in the SSNs (Figure 6), it can be seen that CsaB-like pyruvyltransferases occurred mainly in the phyla of *Firmicutes* and *Cyanobacteria*; Pvg1p-like pyruvyltransferases occurred mainly in the phyla of *Proteobacteria* and *Firmicutes*; and WcfO-like pyruvyltransferases occurred almost exclusively in the phyla of *Bacteroidetes, Proteobacteria*, and *Firmicutes*. In most cases, these different phyla separated nicely into different clades. For the SSN of Pvg1p-like pyruvyltransferases, however, there were two clusters where *Proteobacteria* were heavily mixed with *Firmicutes* and *Bacteroidetes*, and in the SSN of WcfO-like pyruvyltransferases, *Proteobacteria* were found to be heavily mixed with *Bacteroidetes*. Such mixed sequence populations might occur because of the high rates of lateral gene transfer among *Proteobacteria* [173]. Looking across all three SSNs, there was typically only one major cluster for each phylum. The only exception was pyruvyltransferase sequences from *Firmicutes*, which showed multiple big clusters in all three SSNs, indicating that *Firmicutes* might carry multiple types of pyruvyltransferases.

Analysing the functionally characterized pyruvyltransferase CsaB in the context of its surrounding sequence space showed the enzyme to be a typical representative of the biggest cluster (*Firmicutes*) in the CsaB-like SSN. The same goes for WcfO, which was also found within the biggest cluster (*Bacteroidetes* and *Proteobacteria*) of the WcfO-like SSN. Pvg1p, on the other hand, was found at the border of a minor *Ascomycota* clade in the Pvg1p-like network and, therefore, cannot be considered a typical representative of this network. It is interesting to note, however, that Pvg1p is a pyruvyltransferase from the fungal phylum of *Ascomycota*, but the sequence search based on Pvg1p resulted mainly in bacterial sequences from *Proteobacteria* and *Firmicutes*, rather than other fungal sequences.

In addition to CsaB, Pvg1p, and WcfO, this review further discussed 48 putative pyruvyltransferases, and about half of their corresponding amino acid sequences were present within the calculated SSNs. Possible reasons for this incomplete recovery of sequences in the SSNs were the lack of genome sequencing data, missing or faulty taxonomic annotation of sequences, and the fragmentary coverage of the pyruvyltransferase sequence space in the performed SSN analysis.

Note that this study refrained from removing sequences showing 100% sequence identities (possible duplicates), meaning that the utilized datasets included all currently known pyruvyl-transferase entries found under the given search parameters on UniProtKB. It is inevitable that this leads to a possible bias in sequence counts towards organisms that are more heavily sequenced than others, but at the same time, it guarantees the representation of the full taxonomic distribution of pyruvyltransferases. From these datasets, the phyla *Firmicutes*, *Proteobacteria*, *Cyanobacteria*, and *Bacteroidetes* were found to be the phyla where pyruvyltransferases are most common.

From this study, it is evident that the pyruvyltransferase sequences available in public databases are extremely diverse, and without the availability of further biochemically characterized pyruvyltransferases, predictions of pyruvyltransferases based on amino acid sequences have to be interpreted with care.

## 6. Discussion

Pyruvyltransferases are a widespread but little investigated class of carbohydrate-active enzymes, which transfer a pyruvate moiety from a PEP donor to various monosaccharide targets (Table 1). This leads to a wealth of glycoconjugates carrying this modification. Pyruvylation can be found in almost all classes of glycoconjugates—including EPS, CPS, CA, LPS, LOS, SCWP, and *N*-glycans—occurring in bacteria, algae, and yeast, but not in humans. Importantly, pyruvylation imparts an anionic character to the glycoconjugates, which is pivotal to many biological functions. Described functions include the influence on the viscosity of the EPS, bacterial symbiosis with plants [18,28,46], immunostimulatory effects (mostly of CPSs [7,93]), employment of sialylation-like properties in human-type oligosaccharides [198], and cell wall anchoring relying on the Pyr-β-d-ManNAc epitope [14,99,104,195], to name a few. However, learning more about the biological significance of pyruvylated glycoconjugates and delineating a possible association between the position of pyruvylation and functionality are remaining challenges for future research.

Regrettably, for most of the described pyruvylated glycoconjugates, the genetic determinants of the modification are unknown because of missing genome sequencing data of the respective organisms. Given the widespread occurrence and the importance of sugar pyruvylation in nature, there is a high interest in the research community to identify pyruvyltransferases and gain insight into the mechanism of pyruvylation, especially with regard to the high potential to reveal novel functions and drug target points.

Up until now, three orthologous pyruvyltransferases have been biochemically investigated [7,111,161]. However, they do not show any close sequence relationship (compare with Figure 6). This finding might point towards a convergent evolution of pyruvyltransferases or a very high evolutionary rate that underlines the high sequence variability present in this enzyme class. The SSNs established within the frame of this review indicate that the described sequence space around the three hitherto characterized sequences was not sufficient to cover the whole extent of sequence variation of pyruvyltransferases. Based on the currently available sequence information, pyruvyltransferases mainly occur in bacterial phyla of *Firmicutes*, *Proteobacteria*, *Cyanobacteria*, and *Bacteroidetes*, and to a lesser extent in eukaryotic species.

Given the relentless spread of antibiotic-resistant organisms, new chemotherapeutic strategies to overcome infections could be based on intervening in the mechanisms of pyruvylation, an enzymatic modification detected in almost all classes of cell envelope glycoconjugates.

## Figures and Tables

**Table 1:** Pyruvylated saccharides and positions of pyruvate found in the organisms described in this review.

Organism	Saccharide (Stereochemistry)	Position of Pyr	Biological Significance *		References
			Pyr-Saccharide	Pyr-Glycoconjugate	
Exopolysaccharides					
*Xanthomonas* spp.	Man (β)	4,6		Plant pathogenesis	[18,28,32]
*Xanthomonas campestris*	Man (β)	4,6	Virulence		[18,28,32]
*Sinorhizobium meliloti*	Glc (?)	?	Glycan polymerization and export		[41]
*Rhizobium leguminosarum*	Gal; Glc (β)	4,6	Signalling		[46]
*Agrobacterium* sp. ZX09	Glc (?)	?		Plant symbiosis	[47]
*Escherichia coli*	Gal (α)	4,6		Biofilm	[32,50]
*Burkholderia cepacia*	Gal (α)	4,6		Pathogenesis	[53]
*Pseudomonas strain* 1.15	Gal (α)	4,6			[54]
*Enterobacter amnigenus*	Man (α)	4,6			[55]
*Inquilinus limosus*	Man (α); Glc (β)	4,6		Pathogenesis	[56]
*Azorhizobium* *caulinodans*	Gal (α)	4,6		Plant symbiosis	[59]
*Cobetia marina* DSMZ 4741	Kdo (α)	7,8			[60]
*Pediococcus pentosaceus*	hexose (?)	?			[61]
*Erwinia futululu*	Gal (α)	4,6		Pathogenesis	[63]
*Agrobacterium radiobacter* ATCC 53271	Glc (α)	4,6			[64]
*Methylobacterium* sp.	Gal (α)	4,6			[65]
*Alteromonas macleodii* subsp. *fijiensis*	Man (?)	4,6			[66,67]
Capsular polysaccharides					
*Streptococcus pneumoniae*	Gal (α)	2,3	Immunostimulant		[74]
*Acinetobacter baumannii*	GalNAc (α)	4,6		Virulence	[78]
*Klebsiella pneumoniae*	GlcA (β)	2,3	Stimulation of immune system		[79]
*Klebsiella* serotype K14	Glc (β)	4,6			[82]
*Klebsiella* serotype K12	Gal*f* (α)	5,6			[83]
*Klebsiella* serotype K70	Rha (α)	3,4			[84]
*Klebsiella* serotype K64	Gal (β)	4,6			[85]
*Klebsiella* serotype K46	Man (β)	4,6			[86]
*Klebsiella* serotype K33	Gal (β)	3,4			[87]
*Klebsiella* serotype K3	Man (α)	4,6		Immunogenicity; binding to human mannose receptor	[89]
*Bacteroides fragilis*	Gal (β)	4,6		Virulence	[7]
*Rhodococcus equi* serotype 4	RhoAc (α)	7,9		Pathogenesis	[95]
Non-classical secondary cell wall polymers					
*Bacillus anthracis*	ManNAc (β)	4,6	Binding of SLH-proteins		[102]
*Bacillus cereus*	ManNAc (β)	4,6	Binding of SLH-proteins		[17]
*Paenibacillus alvei*	ManNAc (β)	4,6	Binding of SLH-proteins		[15]
*Lysinibacillus sphaericus* CCM 2177	ManNAc (β)	4,6	Binding of SLH-proteins		[121]
*Paenibacillus (Bacillus) polymyxa*	ManNAc (β)	4,6			[130]
Secondary cell wall glycopolymers					
*Promicromonospora citrea* 665^T^	Kdn (α); Gal (α)	7,9; 4,6			[131]
*Promicromonospora* sp. VKM Ac-1028	GlcA (β)	2,3			[131]
*Nocardiopsis metallicus* VKM Ac-2522T	Rib-ol-P (?)	2,4			[133]
*Nocardiopsis halotolerans*	GalNAc (?)	4,6			[133]
*Brevibacterium iodinum* VKM Ac-2106	Man-ol; GalNAc (α)	4,5; 4,6			[135]
Lipopolysaccharides and lipooligosaccharides					
*Pseudomonas stutzeri*	Glc (β); GlcNAc (β)	4,6	Glycoconjugate biosynthesis		[4]
*Providencia alcalifaciens*	GlcNAc (α)	4,6		Pathogenesis	[141]
*Shigella dysenteriae*	Man (β)	4,6		Pathogenesis	[145]
*Proteus mirabilis* O16	GalNAc (α)	4,6			[147]
*Raoultella terrigena*	Man (β)	4,6			[146]
*Cobetia pacifica* KMM 3878	Gal (β)	3,4			[148]
Mycobacterial glycolipids					
*Mycobacterium smegmatis*	Me-Glc (α)	4,6			[150]
*Mycobacterium avium-Mycobacterium intracellulare* (MAI) serovar 8	Me-Glc (α)	4,6	Immunostimulant		[151]
MAI serovar 21	Glc (α)	4,6			[151]
Pyruvylated glycoconjugates in eukaryotes					
*Aplysia kurodai*	Gal (β)	3,4			[152]
*Schizosaccharomyces pombe*	Gal (β)	4,6	Charge effect (mimic of sialylation?)		[9]
Red algae	Gal (β)	4,6		Immunostimulant	[10,13,161,162]
Green algae	Gal (?)	3,4		Anticoagulant	[11,12]
*Microciona prolifera*	Gal (β)	4,6		Species-specific cell reaggregation	[163]

* The biological significance is given, when known. SLH: S-layer homology.

## Abbreviations

4,6Pyr	4,6-ketal-pyruvate
AADGal*p*	α-d-2-*N*-acetylamido-4-amino-galactopyranose
Ac	acetyl
Am	aecetamido
CA	colonic acid
Cm	carbamoyl
CPS	capsular polysaccharide
CSDB	Carbohydrate Structure Database
Cys	cysteine
D	aspartic acid
DPNH	reduced diphosphopyridine nucleotide
EPS	exopolysaccharide
ER	endoplasmic reticulum
Ery-ol	erythritol
ESI-MS	electrospray ionization mass spectrometry
*f*	furanosidic
Fuc	fucose
Gal	galactose
GalN	galactosamine
GalNAc	*N*-acetylgalactosamine
GalNAcA	α-*N*-acetyl-d-galactosamine uronic acid
GC	gas chromatography
Glc	glucose
GlcA	glucuronic acid
GlcNAc	*N*-acetylglucosamine
GOESY	gradient nuclear Overhauser and exchange spectroscopy
GT	glycosyltransferase
H	histidine
HMBC	hetero multiple bond correlation
HMM	Hidden Markow Model
HPLC	high-performance liquid chromatography
Kdn	2-keto-3-deoxy-d-*glycero*-d-*galacto*-nononic acid
Kdo	3-deoxy-d-*manno-*oct-2-ulosonic acid
L	leucine
LOS	lipooligosaccharide
LPS	lipopolysaccharide
LTA	lipoteichoic acid
MAI complex	Mycobacterium avium-Mycobacterium intracellulare complex
Man	mannose
Man-ol	mannitol
ManNAc	*N*-acetylmannosamine
Me	methyl
MOPDG	4,6Pyr-*O*Me-β-d-Gal*p*
MurNAc	*N*-acetylmuramic acid
NOESY	nuclear Overhauser and exchange spectroscopy
*p*	pyranosidic
P	phosphate
PEP	phosphoenolpyruvate
PEtN	phosphoetanolamine
pNP	*para*-nitrophenyl
Pyr	pyruvate
pyrGal-ase	pyruvylated galactose-releasing enzyme
QuiNAc	*N*-acetylquinovosamine
R	arginine
Rha	rhamnose
Rho	5-amino-3,5-dideoxynonulosonic (rhodaminic) acid
Rib-ol	ribitol
ROESY	rotating frame Overhauser enhancement spectroscopy
RP	reversed phase
RU	repeating unit
SAP	plasma glycoprotein serum amyloid P component
SCWP	secondary cell wall glycopolymer
Ser	serine
SLH	surface layer homology
sp.	species
spp.	species (*plural*)
TA	teichoic acid
undp-P	undecaprenylphosphate
WTA	wall teichoic acid
